# PB-LNet: a model for predicting pathological subtypes of pulmonary nodules on CT images

**DOI:** 10.1186/s12885-023-11364-6

**Published:** 2023-10-03

**Authors:** Yuchong Zhang, Hui Qu, Yumeng Tian, Fangjian Na, Jinshan Yan, Ying Wu, Xiaoyu Cui, Zhi Li, Mingfang Zhao

**Affiliations:** 1 Department of Medical Oncology, the First Hospital of China Medical University, NO.155, North Nanjing Street, Heping District, Shenyang, Liaoning Province 110001 China; 2 College of Medicine and Biological Information Engineering, Northeastern University, NO. 3-11, Wenhua Road, Heping District, Shenyang, 110819 Liaoning Province China; 3 Network Information Center, China Medical University, NO.77 Puhe Road, Shenbei New District, Shenyang, Liaoning Province 110122 China; 4 Phase I Clinical Trails Center, the First Hospital of China Medical University, 210 1st Baita Street, Hunnan Distriction, Shenyang, Liaoning Province 110101 China; 5 Key Laboratory of Intelligent Computing in Medical Image, Ministry of Education, Shenyang, China

**Keywords:** Pulmonary nodules, Deep learning, Classification, CT, Lung cancer

## Abstract

**Objective:**

To investigate the correlation between CT imaging features and pathological subtypes of pulmonary nodules and construct a prediction model using deep learning.

**Methods:**

We collected information of patients with pulmonary nodules treated by surgery and the reference standard for diagnosis was post-operative pathology. After using elastic distortion for data augmentation, the CT images were divided into a training set, a validation set and a test set in a ratio of 6:2:2. We used PB-LNet to analyze the nodules in pre-operative CT and predict their pathological subtypes. Accuracy was used as the model evaluation index and Class Activation Map was applied to interpreting the results. Comparative experiments with other models were carried out to achieve the best results. Finally, images from the test set without data augmentation were analyzed to judge the clinical utility.

**Results:**

Four hundred seventy-seven patients were included and the nodules were divided into six groups: benign lesions, precursor glandular lesions, minimally invasive adenocarcinoma, invasive adenocarcinoma Grade 1, Grade 2 and Grade 3. The accuracy of the test set was 0.84. Class Activation Map confirmed that PB-LNet classified the nodules mainly based on the lungs in CT images, which is in line with the actual situation in clinical practice. In comparative experiments, PB-LNet obtained the highest accuracy. Finally, 96 images from the test set without data augmentation were analyzed and the accuracy was 0.89.

**Conclusions:**

In classifying CT images of lung nodules into six categories based on pathological subtypes, PB-LNet demonstrates satisfactory accuracy without the need of delineating nodules, while the results are interpretable. A high level of accuracy was also obtained when validating on real data, therefore demonstrates its usefulness in clinical practice.

## Introduction

The widespread use of computed tomography (CT) has contributed to a remarkable increase in the detection rate of pulmonary nodules. Pulmonary nodules refer to lung shadows of no more than 3 cm in diameter, round-like, with increased density, which may be solid or sub-solid [1–3]. They can be further classified into benign and malignant lesions based on pathology. The benign lesions include inflammation and fibrosis, etc., which do not require anti-tumor therapy; For malignant lesions, interventions such as surgery, ablation and radiation therapy are needed. In 2021, WHO classifies tumors of the same origin into atypical adenomatous hyperplasia (AAH), adenocarcinoma in situ (AIS), minimally invasive adenocarcinoma (MIA) and invasive adenocarcinoma (IA) according to their progression from dysplasia to in situ lesions then to invasive lesions. AAH and AIS may together be described as precursor glandular lesions. Invasive non-mucinous adenocarcinoma is one of the most common types of adenocarcinomas, for which International Association for the Study of Lung Cancer (IASLC) established a grading system. Grade 1 refers to lepidic predominant tumor; Grade 2 refers to acinar or papillary predominant tumor. Both contains less than 20% of high-grade patterns, which include solid, micropapillary, or complex gland. Grade 3 contains any tumor with 20% or more of high-grade patterns [4]. This grading system has a higher prognostic predictive value than other systems and training models [5]. Different pathological subtypes of pulmonary nodules require different interventions and have different prognosis, so the accurate classification is important for clinical decision.

The diagnosis of pulmonary nodules relies on surgical and non-surgical biopsies such as bronchoscopy and Percutaneous transthoracic needle biopsy [2]. In clinical practice, however, there are still difficulties in making an accurate diagnosis of lung nodules. Therefore, the CT appearance of lung nodules can be used as a reference to aid diagnosis and guide clinical decision making. The clinician is required to evaluation of the nodules' appearance and internal features, including their sizes, shapes, margins, densities and structures, to obtain a more accurate diagnosis and classification. However, manual assessment of lung nodules is subjective and only a rough differentiation of benign and malignant pulmonary nodules. This may result in a less accurate diagnosis and an inability to classify pathological subtypes. Radiomics can extract a large amount of image features and is used to address many clinical problems. However, radiomics requires manual delineation of the region of interest (ROI), adding additional workload. In addition, the features are got through manual selection, which has some limitations and is difficult to achieve better results.

Deep learning has been widely applied to the analysis of medical images [6–9], and the commonly used method is convolutional neural networks (CNN), which include AlexNet, VGGNet, GoogLeNet, ResNet, ResNext and other common structures [10–14]. Compared to radiomics, deep learning is more automated, eliminating the need for manual delineation of ROIs and allowing for automatic feature selection. However, there are limitations in the current research on the classification of lung nodules. At present, deep learning studies on lung nodules focus on benign and malignant differentiation, with only a few studies on multiple classifications and grading of adenocarcinomas. The objective of this study was to propose a novel framework, PB-LNet, for identifying pathological subtypes of lung nodules using preoperative lung CT images, and eliminating the requirement of outlining ROIs, which holds potential for guiding clinical management.

## Materials and methods

### Patient data

**Fig. 1:**
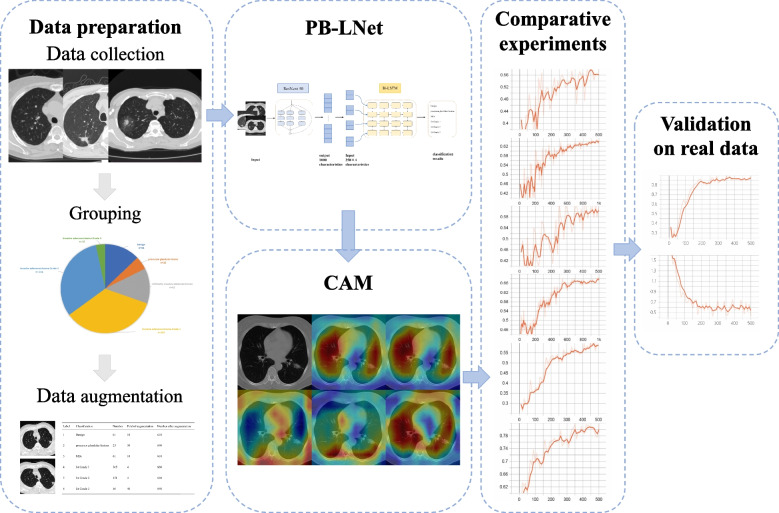
The overall flow of the study

The overall flow of this study is shown in Fig. 1. We retrospectively collected the information of patients with pulmonary nodules treated by surgery in Department of Thoracic Surgery, The First Affiliated Hospital of China Medical University from July 2014 to February 2019. The inclusion and exclusion criteria were like following. Inclusion Criteria: (1) The patients’ age should be at least 18 years. (2) The long diameters of the nodules range from 3–30 mm and the nodules underwent surgical resection. (3) Complete CT images within 30 days before surgery are available. (4) All patients had definite pathological diagnosis and complete clinical data. Exclusion criteria: (1) Poor image quality, such as respiratory or other movement artefacts. (2) Patients with disease such as chronic pulmonary disease or acute pneumonia affecting the observation of the nodule. (3) Patients diagnosed with lung cancer but not adenocarcinoma. (4) Patients with invasive mucinous adenocarcinoma or rare types such as fetal adenocarcinoma, intestinal-type adenocarcinoma and colloid adenocarcinoma. (5) Patients with multiple primary cancers.

The following information was collected on the patient: gender, age, personal history such as smoking and family history of tumors. Complete preoperative CT images of the lungs and pathology reports were also collected.

**Fig. 2:**
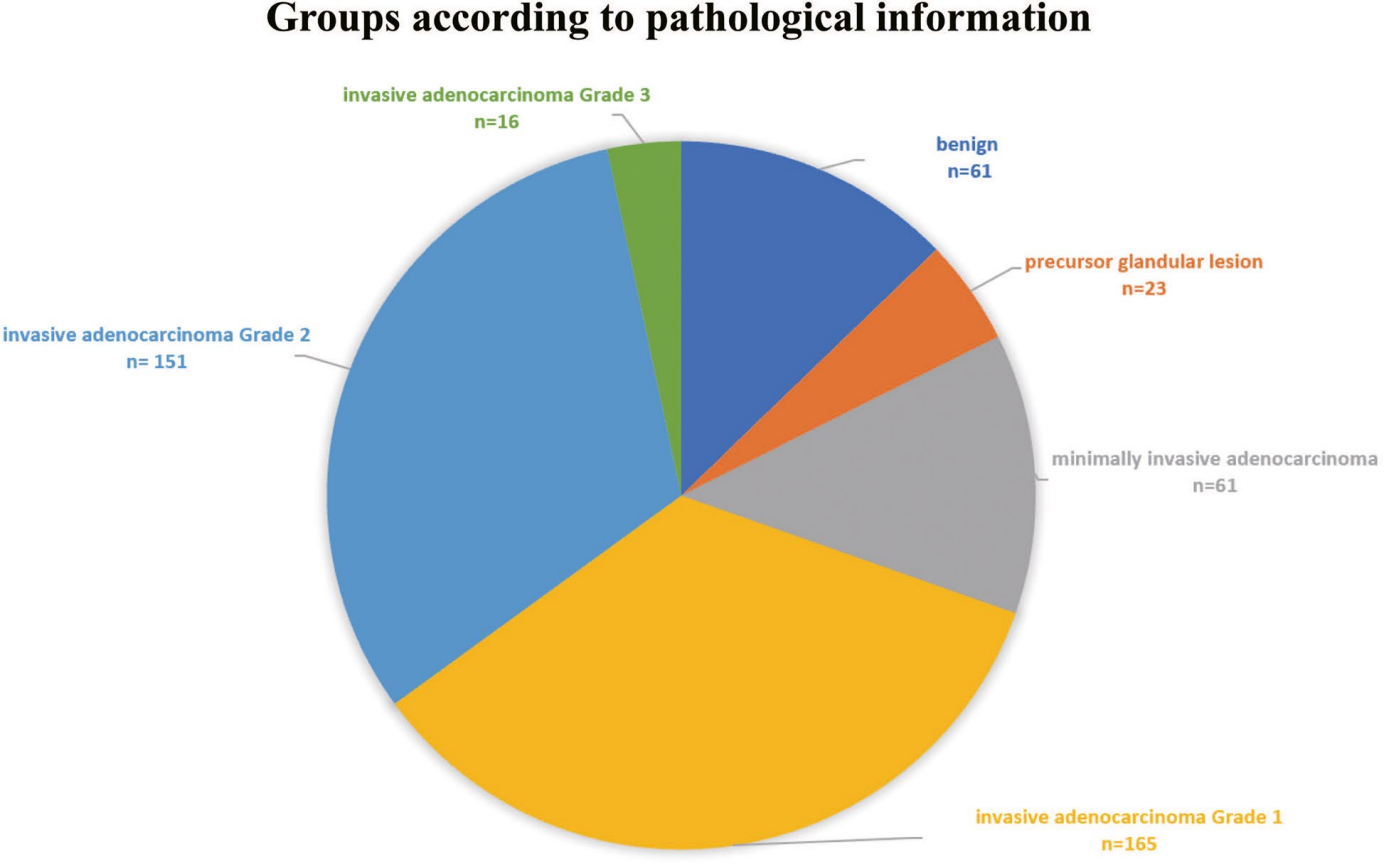
Groups according to pathological information

Based on the pathological information of the lesion, the data was divided into the following six groups: benign lesions, precursor glandular lesions, minimally invasive adenocarcinoma, invasive adenocarcinoma Grade 1, invasive adenocarcinoma Grade 2 and invasive adenocarcinoma Grade 3, as is shown in Fig. 2. Ethics approval was obtained (AF-SOP-07–1.1–01).

### CT image acquisition and image preprocessing

**Fig. 3:**
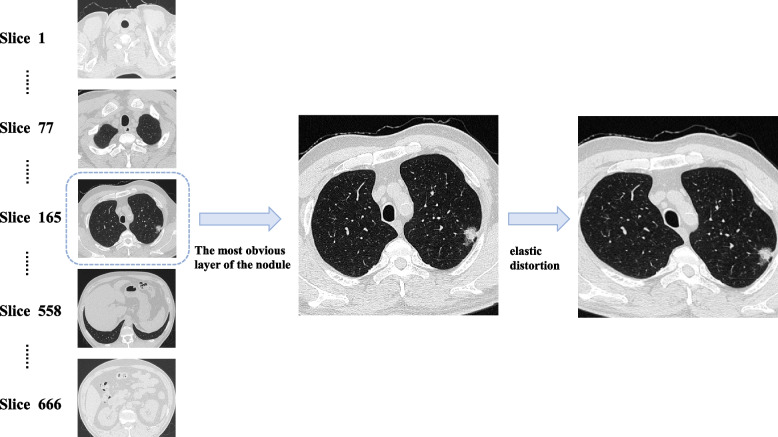
Layer selection and data augmentation. Select the most obvious layer of the nodule on CT images and use elastic distortion for data augmentation

The patients' CTs are mainly obtained using GE and SIEMENS CT scanner. The layers were selected in CT plain images with the window width of 1500HU and window level of—550HU. The most obvious layer of the nodule is selected for analysis (Fig. 3). In order to eliminate the influence of background and other tissues and organs on the training results, we use a image segmentation algorithm based on kmeans clustering to extract the lung image. Based on the clustering results, the finer elements are eroded, and then dilated to include some pixels around the lung to ensure the extraction of a complete lung image. Then, the pixel value of the processed image is normalized to the range of 0–1.

### Data augmentation

Due to the small sample size and the imbalanced data, it is required to enhance the data to avoid overfitting in training [15]. For image data, it is common to use affine transformations such as rotation, displacement and zoom for data augmentation, but these methods may lose some of the information in the image. Here we use elastic distortion, which allows the network to learn the distortion features and the attributes that need to be learned in order to improve the performance of the model. The selected CT image is first applied affine transformation to obtain the transformed image. Next, a random displacement field is created and the image is deformed. Δx and Δy represent the displacement of each pixel point in the x and y directions respectively, with a displacement range of (-1, 1). The standard deviation of the Gaussian function is σ. The scale factor α controls the intensity of the deformation, and by multiplying the convolved displacement field with α, the displacement field of the elastic distortion can be obtained. The effect of the elastic distortion is shown in Fig. 3, and the number of images before and after data augmentation in each group is shown in Table 1.

**Table 1:** Number of cases in each group before and after data augmentation

Label	Classification	Number	Fold of augmentation	Number after augmentation
1	Benign	61	10	610
2	precursor glandular lesions	23	30	690
3	MIA	61	10	610
4	IA Grade 1	165	4	660
5	IA Grade 2	151	4	604
6	IA Grade 3	16	40	640

*MIA* Minimally invasive adenocarcinoma, *IA* invasive adenocarcinoma

### PB-LNet

The enhanced data was randomly divided into training set, validation set, and test set at the ratio of 6:2:2. The training set was used to train PB-LNet, while the validation set was used to adjust the parameters. The test set was used to evaluate the performance.

PB-LNet consists of ResNext50 and a Bi-LSTM with 4 hidden layers. The batch size, the initial learning rate and the number of iterations were set to 8, 0.01 and 500. L2 regularization was used to prevent overfitting. The ImageNet was utilized for the pre-training. All parameters of the layers except for the last one were frozen and 1000 features were output from the last layer.

The 1000 features were equally divided into 4 groups and were fed into the Bi-LSTM, which has 4 hidden layers and 1000 nodes.

The accuracy (ACC) was used as an index to assess the performance of the classification model, which is calculated as the ratio of correct classification to the total predicted classification.

Loss function can be used to estimate the difference between the predicted value f(x) and the real value Y. When training the network, the prediction performance can be gradually optimized by adjusting the parameters until the best prediction performance is achieved and the loss function tends to be stable. Different loss functions are used for different learning problems. For regression problems, the mean square error loss function is generally used. For classification problems in our study, we used the cross entropy loss as the loss function [16].

### Class Activation Mapping

Class Activation Mapping (CAM) can highlight the regions used for image classification, which allows us to interpret the result of the network. The redder color represents the regions of more importance for classification. To explore the interpretability to our model, we performed CAM on the CT images and observed the focus area of the model.

### Comparative experiments

To compare the performance of PB-LNet with other models, a series of comparative experiments were conducted. CNN is the most commonly used framework for image classification [17, 18]. Here, we used CNN models ResNet18 and ResNext50 as contrast and the number of iterations were set to be 500 and 1000 iterations.

To verify that adding Bi-LSTM can lead to a better prediction performance and compare the training outcomes among different CNN combined with Bi-LSTM, ResNet18 and ResNext50 were each combined with a Bi-LSTM containing one hidden layer to construct the networks. These processes were carried out for 500 iterations.

### Validation on real data

To evaluate the clinical utility of PB-LNet, we analyzed 96 CT images without elastic distortion in the test set. ACC was used to evaluate the classification result of the original CT images.

## Result

### Clinical characteristics

**Fig. 4:**
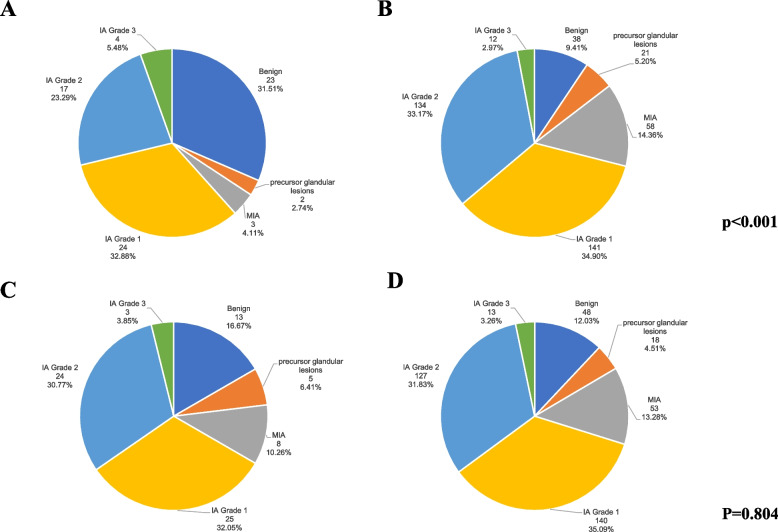
Distribution of pathological subtypes based on patients' smoking and family histories. **A** Distribution of pathological subtypes among patients with smoking history; **B** Distribution of pathological subtypes among patients without smoking history; **C** Distribution of pathological subtypes among patients with family history; **D** Distribution of pathological subtypes among patients without family history

The study included a cohort of 477 patients, comprising 61 cases of benign leisions, 23 instances of precursor glandular lesions, 61 cases of MIA, 165 cases of IA Grade 1, 151 cases of IA Grade 2, and 16 cases of IA Grade 3. The demographic characteristics, including age, gender, smoking history, and family history, were recorded for each patient, and the situation of these variables for each classification is provided in Table 2. The smoking history of patients is significantly associated with the distribution of pathological subtypes (*p* < 0.001), whereas no significant difference in the distribution of pathological subtypes exists between patients with and without a family history (*p* = 0.804). Figure 4 displays the distribution of pathological subtypes among patients with varying smoking histories and family histories in the form of a pie chart.

**Table 2:** Patient clinical information

	Benign (*n* = 61)	precursor glandular lesions (*n* = 23)	MIA (*n* = 61)	IA Grade 1 (*n* = 165)	IA Grade 2 (*n* = 151)	IA Grade 3 (*n* = 16)	p
Gender							0.024
Male	31	6	16	64	50	9	
Female	30	17	45	101	101	7	
Age							< 0.001
Mean (SD)	57.10 (9.37)	61.13 (8.34)	58.82 (8.59)	60.83 (7.77)	62.83 (7.83)	60.19 (4.76)	
Smoking history							< 0.001
Smoker	23	2	3	24	17	4	
Nonsmoker	38	21	58	141	134	12	
Family history							0.804
Have	13	5	8	25	24	3	
None	48	18	53	140	127	13	

*MIA* minimally invasive adenocarcinoma, *IA* invasive adenocarcinoma, *SD* Standard Deviation

### Result of PB-LNet

**Fig. 5:**
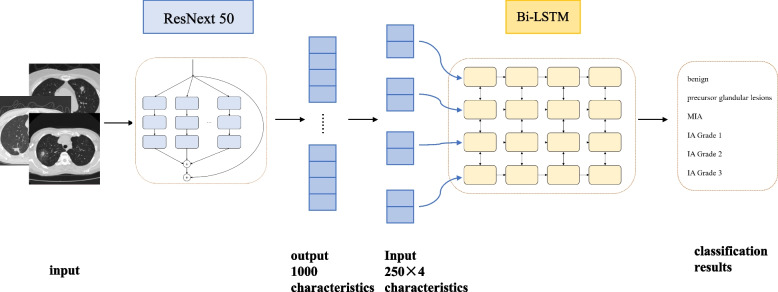
The structure of PB-LNet. PB-LNet consists of ResNext50 and a Bi-LSTM with 4 hidden layers. Input CT images and 1000 features were got. Then equally divided them into 4 groups and fed into the Bi-LSTM. Finally the classification results are output

The structure of PB-LNet is shown in Fig. 5. Across multiple pre-experiments, the hyperparameters were set as following: batch size = 8, initial learning rate = 0.01, the number of iterations = 500 and weight decay = 5e-5. Because of the small training data, pre-trained weights were used for ResNext50. The training and testing procedures is illustrated in Table 3. The ACC of the training set, the validation set, and the test set is stable, which indicated that the model training was completed. Ultimately, the ACC of the training set, the validation set, and the test set are 0.99, 0.80 and 0.84 respectively. The accuracy of each individual class is shown in Table 4. PB-LNet demonstrated satisfactory ACC not only in the overall performance, but also in accurately predicting each individual class.

**Table 3:** Training process of PB-LNet

number of iterations	ACC (training set)	ACC (validation set)	ACC (test set)
100	0.91	0.68	0.71
200	0.98	0.74	0.77
300	0.99	0.76	0.82
400	0.99	0.78	0.83
500	0.99	0.80	0.84

*ACC* accuracy

**Table 4:** ACC for each individual class

Label	Classification	Number of images (training set)	ACC(training set)	Number of images (test set)	ACC (test set)
1	Benign	462	1.00	165	0.83
2	precursor glandular lesions	372	1.00	124	0.94
3	MIA	407	0.99	165	0.85
4	IA Grade 1	490	0.99	160	0.80
5	IA Grade 2	430	0.99	140	0.79
6	IA Grade 3	451	1.00	41	0.90

*ACC* accuracy, *MIA* minimally invasive adenocarcinoma, *IA* invasive adenocarcinoma

### Image visualization

**Fig. 6:**
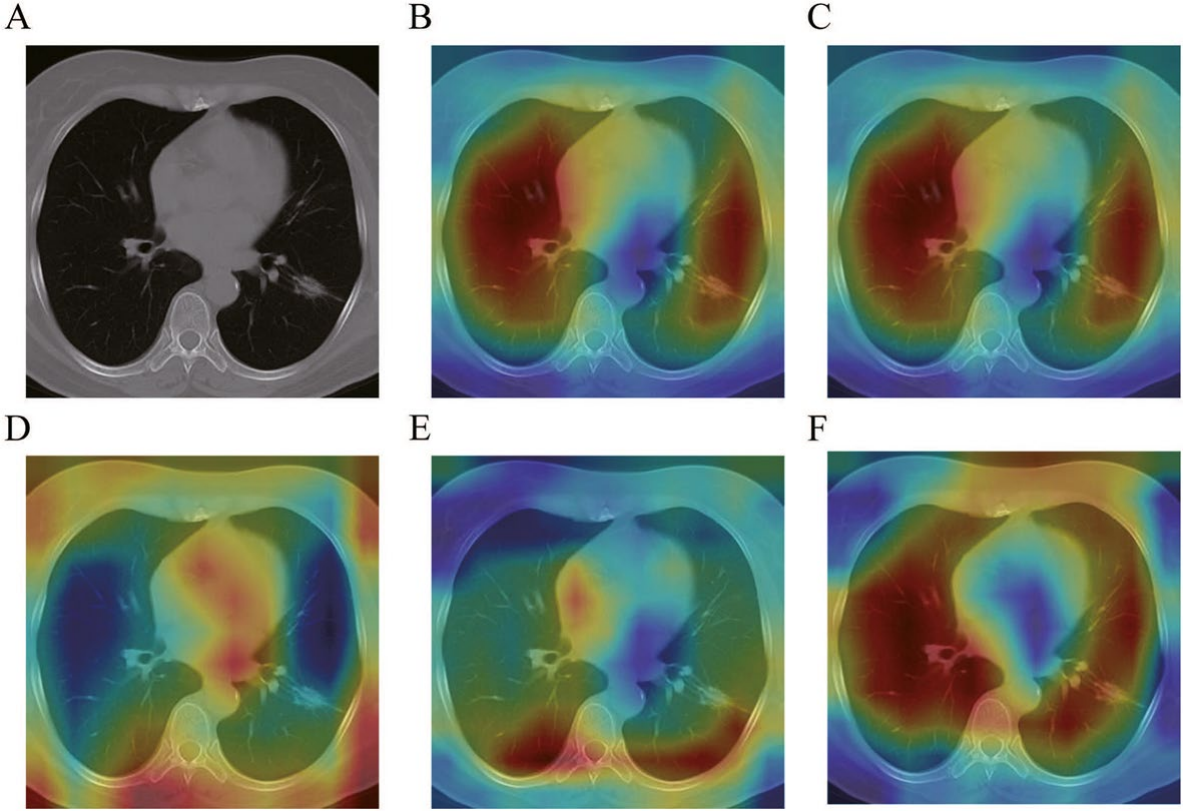
Visualization using CAM to interpret the result. The redder color represents the regions of more importance for classification. Same as actual situation in clinical practice, the classification by PB-LNet is based mainly on the lung. **A** Original image; **B** Overall visualization result; **C** visualization result of input feature Group 1 (feature 1–250); **D** visualization result of input feature Group 2 (feature 251–500); **E** visualization result of input feature Group 3 (feature 501–750); **F** visualization result of input feature Group 4 (feature 751–1000)

Deep learning models have high accuracy but poor interpretability [19]. For interpretation of the results, a Class Activation Map was applied for visualization. The CAM results indicated that our network classified the nodules mainly based on the lungs in CT images, which is in line with the actual situation in clinical practice (Fig. 6). In the pre-experiment, we have used CT images without background removal for training. Although the best accuracy was obtained, the CAM results suggested that it focused mainly on the background region, which is not compatible with the training task.

### Comparative experiments

**Fig. 7:**
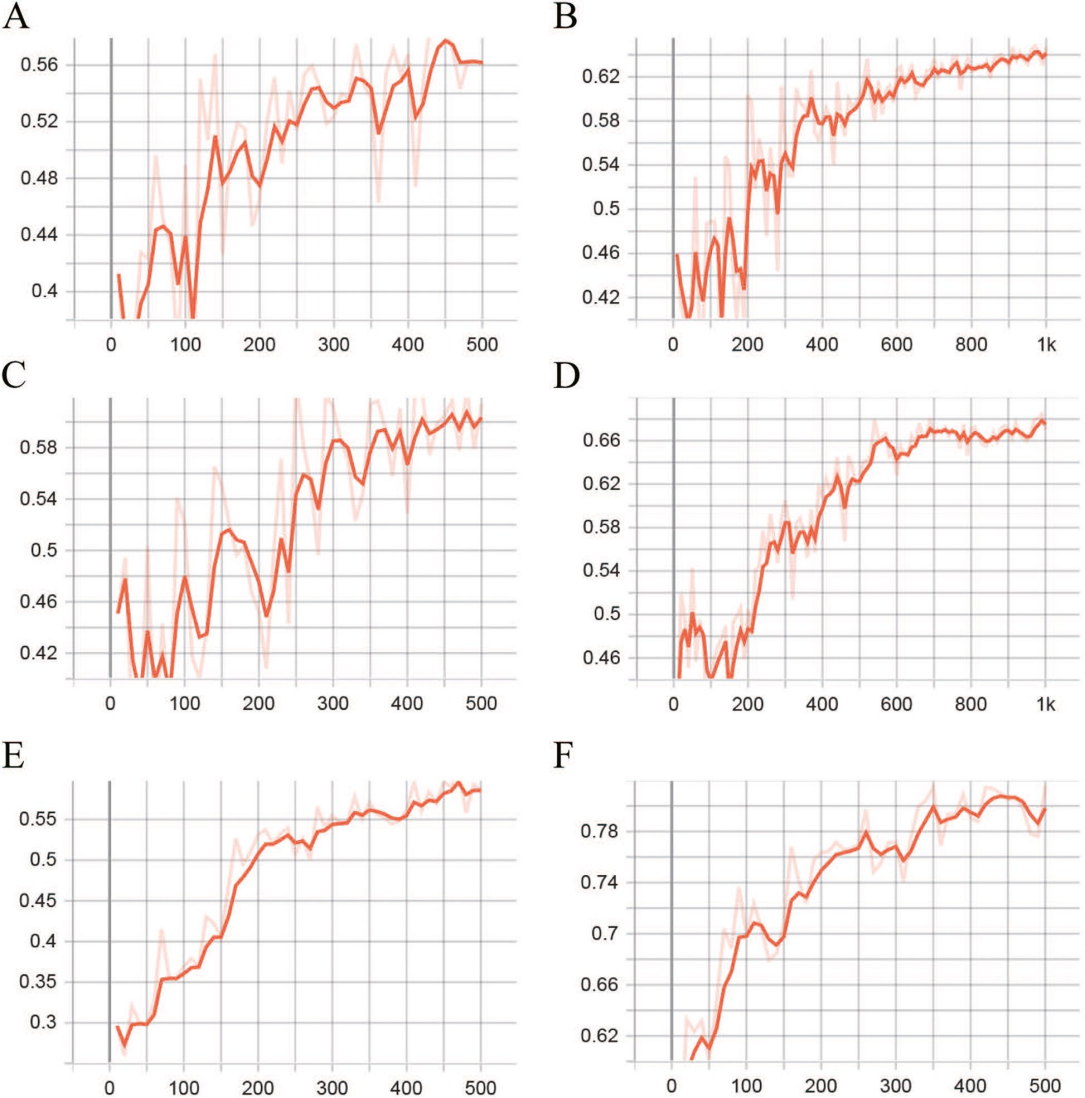
The accuracy of the test set for each network in comparative experiments. **A** ResNet18, number of iterations = 500; **B** ResNet18, number of iterations = 1000; **C** ResNext50, number of iterations = 500; **D** ResNext50, number of iterations = 1000; **E** ResNet18 + Bi-LSTM (with 1 hidden layer), number of iterations = 500; **F** ResNext50 + Bi-LSTM (with 1 hidden layer), number of iterations = 500

To explore the superiority of PB-LNet, we trained different networks, evaluated the results and compared them with our model. The results are shown in Fig. 7 and Table 5.

**Table 5:** Summary of different network structures

Network Structure	Hidden layer of Bi-LSTM	number of iterations	ACC
ResNet18	-	500	0.56
	-	1000	0.65
ResNext50	-	500	0.61
	-	1000	0.67
ResNet18 + Bi-LSTM	1	500	0.59
ResNext50 + Bi-LSTM	1	500	0.82
	4	500	0.84

*ACC* accuracy

As medical image classification is mainly based on convolutional neural networks currently, we explored the capabilities of pre-trained ResNet18 and ResNext50 in nodule classification. When the number of iterations was set to 500, the ACC of the test set was 0.56 and 0.61 respectively, but from the results (Fig. 7A and C), it can be seen that the results were yet optimal; so the number of iterations was adjusted to 1000, and the results have reached stability. The final ACC of the test set was 0.65 and 0.67 respectively and the ACC of ResNext50 was higher. Thereafter, we added Bi-LSTM with one hidden layer to ResNet18 and ResNext50, setting the number of iterations to 500, the ACC was 0.59 and 0.82 respectively. From the above experiments, it can be seen that ResNext50 + Bi-LSTM (with 1 hidden layer) already has a great prediction performance, but PB-LNet still made a further improvement in accuracy.

### Validation on real data

**Fig. 8:**
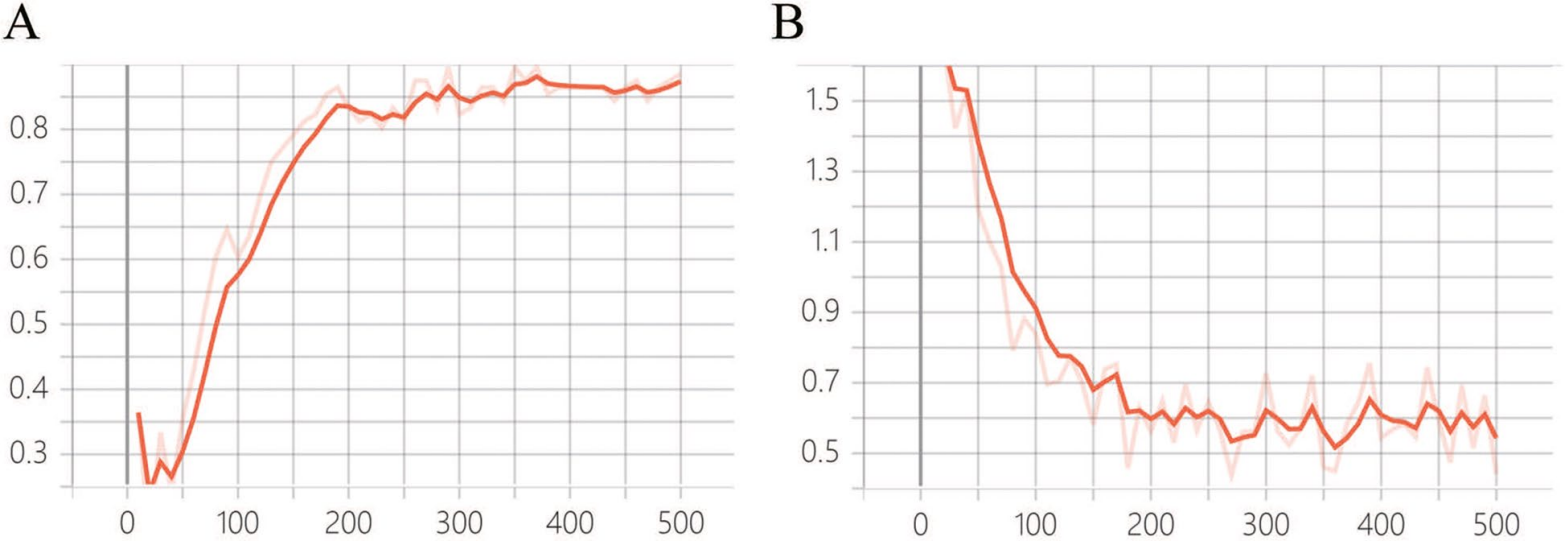
Result of real data validation. The result has reached stability with an ACC of 0.89. **A** Accuracy; **B** Loss function

To evaluate the effect of PB-LNet for nodule classification in original lung CT images without elastic distortion, 96 images from the test set without data augmentation were analyzed. The ACC was up to 0.89, as shown in Fig. 8, representing that the model had good clinical utility.

## Discussion

The incidence and mortality rates of lung cancer are rank at the top among all malignant tumors, the 5-year survival rate of which is less than 20%, poses a huge threat to human health. If early diagnosis and treatment of lung cancer is achieved, the survival rate can be greatly improved. Lung nodule is an important early manifestation of lung cancer and the accurate diagnosis and classification of them plays an important role in clinical management. In this study, we built a model based on deep learning aimed at accurately classifying lung nodules and guiding clinical decisions. ACC of up to 0.84 was achieved in classifying lung nodules into six categories, while visualization with CAM was used to interpret the results of the training. To confirm their practical use in the clinic, we analyzed them in real CT without elastic distortion and achieved an ACC of up to 0.89.

Currently, the clinical judgment of pulmonary nodules relies on the physician's assessment of internal and external features such as size, margins and density of the nodules. However, this method is subjective and may result in a less accurate diagnosis. In addition, in order to make a definitive diagnosis, patients are often required to undergo reexaminations, the interval of which varies from person to person and requires the physician to compare and evaluate the nodules at different time points in order to judge the nature of the nodules. However, the uncertainty of dynamic follow-up is high and changes in CT scan parameters may make comparison more difficult, therefore, our study used single CT images to classify lung nodules according to pathological subtypes.

The diagnosis of lung nodules has critical impact on the subsequent treatment. For benign lesions, long-term follow-up is an option and anti-tumor interventions are generally not required. For malignant lesions, surgery is the most common treatment modality. Patients who are not suitable for surgery may opt for other local treatment such as radiotherapy and ablation. The current standard surgical procedure is lobectomy, as a randomized trial in 1995 showed that for lung cancer staged T1N0, sub-lobar resection resulted in significantly lower survival rates and higher local recurrence rates than lobectomy [20, 21]. In clinical practice, however, patients cannot always tolerate lobectomy. Subsequent studies have shown that there is no survival difference between sub-lobar resection compared to lobectomy for specific patients [22]. Furthermore, sub-lobar resection allows for a reduction in the volume of lung removed, resulting in better preservation of lung function while reducing postoperative complications. Therefore, sub-lobar resection may be considered if certain conditions are met, such as the lung nodule is peripheral, the tumor does not exceed 2 cm in diameter and the pathology is AIS or MIA [20, 23, 24]. Numerous studies have shown that the micropapillary component is a factor contributing to poor prognosis and is associated with an increased risk of recurrence after sub-lobar resection, as well as a higher risk of recurrence and lymph node metastasis [25–27]. However, many stage I lung adenocarcinomas currently opt for direct surgical treatment, making it difficult to have sufficient specimens for preoperative subtype assessment. Our model can predict the pathological subtype of the lesion preoperatively, and is valuable for the choice of surgical methods.

The pathological subtype of invasive non-mucinous adenocarcinoma is instructive for lymph node dissection as well. Currently, lymph node sampling rather than conventional lymph node dissection is considered for invasive adenocarcinoma with pure ground glass opacity (pGGO) on preoperative imaging and a lepidic pattern on intraoperative frozen section [28]. The deep learning model developed in this study allows preoperative prediction of the pathological subtype of lung nodes and provides an important guide to the extent of lymph node dissection.

For patients with regular follow-up, lung nodules can also be assessed using the model developed in this study to guide the review interval and avoid over-screening. Multiple nodules are also more common in clinical practice, with more than half of patients with pulmonary nodules having multiple nodules [29]. For multiple nodules requiring intervention, the guidelines recommend surgery as the first choice. Priority should be given to the management of major lesions, taking into account secondary lesions, while paying attention to the preservation of lung function. The model provided in this study allows for a more detailed classification of nodules on CT images, assisting in the clinical selection of major lesions and also providing a reference for overall treatment plan.

In recent years, deep learning has been widely applied to the analysis of medical images and is an effective tool for the detection and diagnosis of lung nodules. CNN is often applied to classify CT images, and some common architectures are AlexNet, VGGNet, GoogLeNet, etc. In deep learning, as the number of layers in the network increases, the difficulty of training and optimizing increases, which may cause the accuracy of the network decline, also known as the problem of network degeneration. The Residual Network (ResNet) is characterized by the addition of residual units through a short-circuiting mechanism, which reduces the learning difficulty and solves the degeneration problem of deep networks. It is widely used in areas such as image detection, image recognition and image segmentation for its simplicity and practicality. ResNext is a highly modular network that widens on the basic of ResNet. It turns a single-way convolution into a multi-way convolution with multiple branches, distributing the input to multiple ways, then transforming each way, and finally combining the results of all the branches. In contrast, ResNext has a smaller computational size. In addition, there is also research on image classification using Recurrent Neural Network (RNN) [30]. It can take the previous output and train it together in the next hidden layer, so the output depends not only on the input content, but also on the previous output of the network. However, RNN has the problem of short-term memory, that is to say, short-term memory has a greater impact while long-term memory has a smaller impact, making it difficult to train and unable to handle long input sequences. Long short-term memory (LSTM) is a special kind of recurrent neural network that unit consists of an input gate, an output gate, a forget gate [31]. With such a structure, it is possible to retain the important information in longer sequences of data and ignore the unimportant information, thus solving the problem of RNN short-term memory. Bi-LSTM is a special structure with bi-directional LSTM that can obtain long-term dependent information [32–34]. In the field of medicine, it has been reported that CNN combined with LSTM models can be used for the detection of intracranial hemorrhage and retinopathy, and classification of gastrointestinal diseases [35–37]. In this study, we applied ResNext50 in combination with Bi-LSTM to build a prediction model for the classification of lung nodules on CT images and obtained a more satisfactory result.

Our study also has some limitations. As it is a retrospective study, the CT scan parameters were not entirely consistent. The available data was limited to the period between 2014 and 2019, and it is possible that there have been advancements in CT imaging technology since then. However, we chose to focus on the most apparent layer of the nodules, potentially limiting the impact of certain imaging technology advancements. In addition, only patients underwent surgical resection and the pulmonary nodules were pathologically confirmed were included in this study, so the number of cases of malignant lesions was high, leading to selection bias, for which we applied data augmentation to solve this problem. Furthermore, our data reflect only the specific study population and are not suitable for extrapolation to the screening and external hospital CT images for application. Our model classifies lung nodules into six categories based on pathological subtypes, which has been refined in comparing with current studies. But in clinical practice, there are a few patients with other types of pathological diagnosis, such as squamous carcinoma, adenosquamous carcinoma and invasive mucinous adenocarcinoma, which failed to be included in this study because the number of cases was too small for model training. In addition, the model in this study focuses on the most obvious level of a single nodule and does not require delineation of nodules or ROI, providing a convenient clinical application. However, the presence of multiple nodules at the same level cannot yet be resolved and there is room for further improvement of the model.

## Conclusion

Overall, this study demonstrated a network PB-LNet that can predict the classification of pulmonary nodules on CT images. Compared to previous studies, our classification is more detailed, dividing the nodules into six categories based on pathological subtypes to better determine their prognosis and thus provide reference to clinical management. PB-LNet obtained satisfactory accuracy and did not require delineation of pulmonary nodules, which could largely reduce the workload of physicians. The results of CAM also confirmed that PB-LNet focused on areas similar to those in clinical practice when classifying pulmonary nodules. A high level of accuracy was also obtained when validating on real data. Therefore, PB-LNet has excellent predictive power as well as good clinical application, and the results are interpretable.

## Data Availability

The datasets analysed during the current study are not publicly available due to the privacy of the patients but are available from the corresponding author on reasonable request.

## Abbreviations

AAH: Atypical adenomatous hyperplasia
AIS: Adenocarcinoma in situ
MIA: Minimally invasive adenocarcinoma
IA: Invasive adenocarcinoma
CT: Computed tomography
CNN: Convolutional Neural Network
Bi-LSTM: Bi-directional Long Short-Term Memory
ACC: Accuracy
CAM: Class Activation Mapping
pGGO: Pure ground glass opacity
ResNet: Residual Network
RNN: Recurrent Neural Network
ROI: Region of interest
